# Optimizing preoperative planning for total hip arthroplasty using random forest models to predict stem size and compatibility

**DOI:** 10.1186/s12891-026-10022-9

**Published:** 2026-05-29

**Authors:** Takehiro Kaneoka, Takashi Imagama, Tomoya Okazaki, Yuta Matsuki, Takehiro Kawakami, Kazuhiro Yamazaki, Kei Sasaki, Yoshiyuki Asai, Takashi Sakai

**Affiliations:** 1 Department of Orthopaedic Surgery, Yamaguchi University Graduate School of Medicine, Ube, 755-8505 Japan; 2 Department of Orthopaedic surgery, Yamaguchi Prefectural Grand Medical Centre, Hofu, Yamaguchi, JP Japan; 3 Department of Systems Bioinformatics, Graduate School of Medicine, Yamaguchi University, Yamaguchi, Japan

**Keywords:** femoral dimensions, machine learning, preoperative planning, random forest, total hip arthroplasty

## Abstract

**Background:**

Preoperative planning is essential for optimal outcomes post-total hip arthroplasty (THA). Appropriate stem size and compatibility with femoral dimensions are essential in THA to prevent distal fixation. Previous studies have proposed deep learning-based models with image recognition for preoperative planning to determine the best-fit stem; however, whether size and compatibility are adequately optimized remains unclear. This study developed methods that may assist surgeons in preoperative planning to estimate the stem size and compatibility using supervised machine learning models.

**Methods:**

Two Random Forest (RF) models were developed to estimate the best-fit stem size and stem compatibility with the femoral geometry. Femoral size information measured at 10 locations from computed tomography images of 320 hips was used to train the models. The training data included the sizes and compatibility information as the target variables for supervised learning provided by a simulation software currently used in clinical practice to determine the optimal stem. As part of feature engineering, ratios derived from combinations of the 10 measured values were used as learning data. The size estimation model was designed as a seven-class classification model, whereas the compatibility estimation model was a binary classification model, predicting whether the stem will exclusively fit distally or not. Both models were tested using data from 109 hips of patients who underwent THA. The model performances were assessed using accuracy, F1 score, precision, and recall. Each parameter’s impact was determined using feature importance analysis.

**Results:**

The size and compatibility estimation models trained without ratio information showed accuracies of 89.0% (exact-match: 46.8%) and 85.3%, respectively. When it was included, the models’ performance improved, with the size and compatibility estimation accuracies increasing to 92.7% (exact-match: 39.4%) and 87.2%, respectively. Feature importance analysis highlighted that distal medullary cavity diameter is key in size estimation, while overall femoral dimensions are vital for compatibility estimation.

**Conclusions:**

This study developed models that estimate the two critical factors in stem selection: size and compatibility. These models may support preoperative planning for THA using the Accolade II stem, although further validation is required to establish applicability to other implant systems.

## Background

Preoperative planning is essential for achieving optimal outcomes in total hip arthroplasty (THA). Inadequate stem placement may lead to complications, such as aseptic loosening [1] and accelerated wear [2]. Therefore, selecting the appropriate stem based on femoral dimensions is essential due to the wide range of anatomic variations in femoral structure [3, 4].

Choosing the right stem requires consideration of the following two key parameters: size and compatibility. However, even when the stem size is correctly determined, a poor fit within the medullary cavity can result in distal fixation. This outcome contradicts the intended fixation concept of taper wedge stems, increasing the risk of complications such as poor osseointegration and persistent pain [5].

Recent reports have demonstrated the application of deep-learning models in preoperative planning for THA [6]. However, questions remain about whether such models optimize stem size and compatibility effectively. Additionally, only a few studies have investigated alternative machine learning approaches. Therefore, to address this gap, we developed a prediction model for stem size and compatibility using the Random Forest (RF) algorithm based on the femoral dimensions derived from simulation data.

We hypothesized that a highly accurate model could be constructed using simulation results as training data, eliminating the need for surgical datasets. Our objectives were to create a machine learning model that can predict stem size and compatibility using simulation data and to validate its accuracy using data from patients who underwent THA while identifying factors influencing its predictive performance. In clinical practice, such a model could support surgeons—particularly in settings with limited experience or resources—by providing objective guidance for stem selection, thereby improving consistency and efficiency in preoperative planning.

## Methods

### Study design and ethics statement

This retrospective study’s protocol was approved by the Ethics Committee of Yamaguchi University Graduate School of Medicine (approval number: H2021-166-2), and informed consent was obtained from all participants.

### Patients

**Fig. 1:**
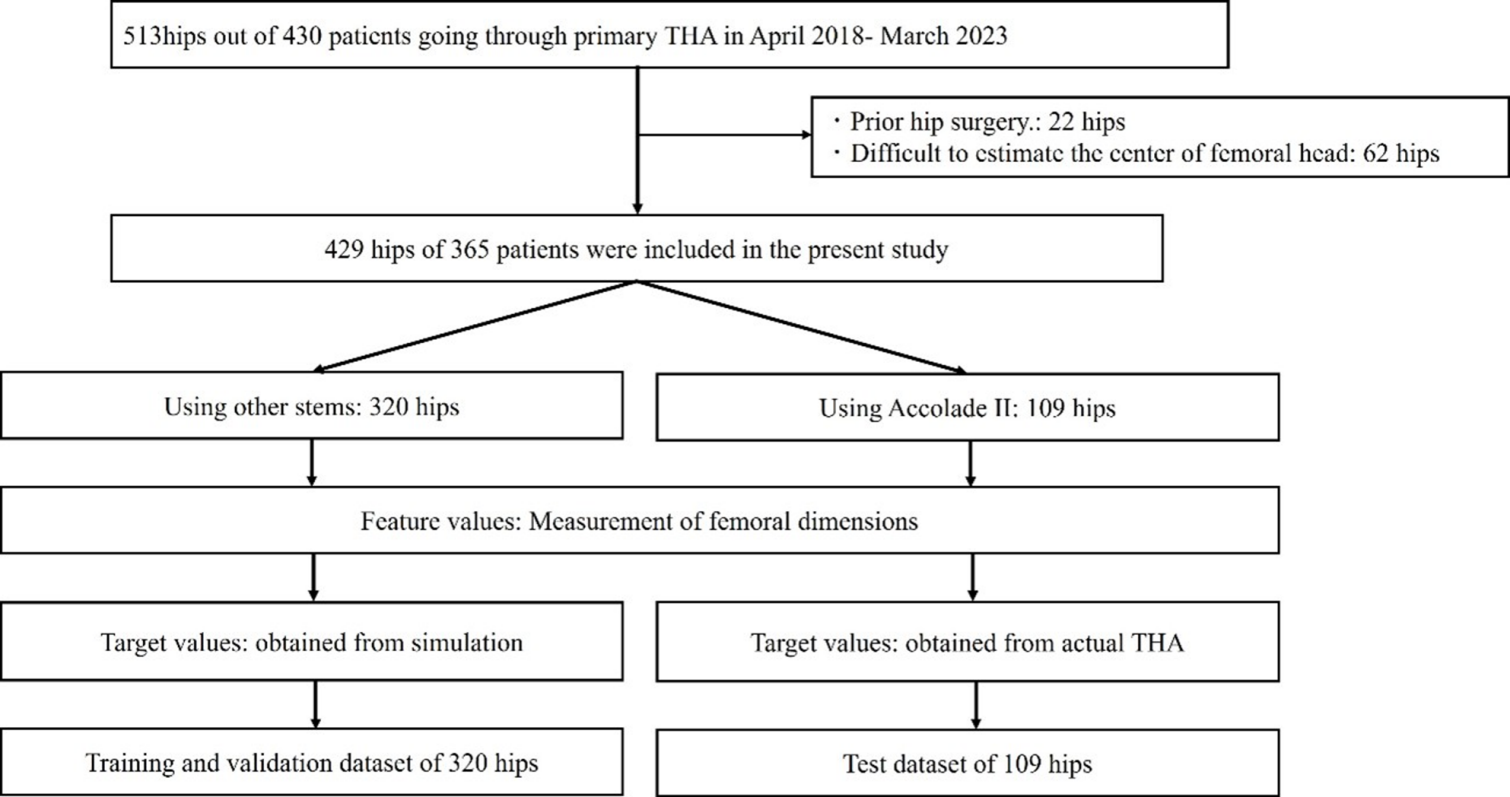
Flow chart of the study population

Overall, 513 hips of 430 patients who underwent primary THA performed by the same surgical team at our institution between April 2018 and March 2023 were evaluated. After excluding 22 hips with a history of contralateral surgery and 62 hips where the femoral head center was difficult to identify (27 with osteoarthritis, 17 with osteonecrosis of the femoral head, 9 with rapidly destructive coxarthrosis, 5 with post-traumatic osteoarthritis, and 4 with rheumatoid arthritis), a total of 429 hips from 365 patients were included in the analysis. They were categorized into a validation dataset consisting of 109 hips that underwent THA using the Accolade II stem (Stryker Orthopedics, Mahwah, NJ, USA) and a training dataset comprising 320 hips treated with other stem types, including Mainstay (Kyocera Medical Corporation, Osaka, Japan; 144 hips), Corail (DePuy Synthes, Warsaw, IN, USA; 141 hips), and Exeter cemented (Stryker Orthopedics, Mahwah, NJ, USA; 5 hips) (Fig. 1). The patient backgrounds did not significantly differ between the two datasets (Table 1).

**Table 1:** Patients background

	Simulation	Underwent THA	
	Training data (320 hips)	Test data (109 hips)	*P* value
Age at surgery (year)	66.1 ± 11.6	67.8 ± 10.6	0.269*
BMI (kg/ m2)	24.3 ± 4.2	24.7 ± 4.2	0.391*
Sex			0.134**
Male	78 (24.4)	19 (17.4)	
Female	242 (75.6)	90 (82.6)	
Dorr			0.585**
A	66 (20.6)	22 (20.2)	
B	234 (73.1)	77 (70.6)	
C	20 (6.3)	10 (9.2)	
Diagnosis			
OA	270 (84.4)	95 (87.2)	
ONFH	23 (7.2)	6 (5.5)	
Other	27 (8.4)	8 (7.3)	
Approach			0.765**
PL	-	87 (79.8)	
m-WJ	-	12 (11.0)	
DAA	-	10 (9.2)	
Stem Size			0.418**
Size 2	25 (7.8)	9 (8.3)	
Size 3	63 (19.7)	27 (24.8)	
Size 4	96 (30.0)	34 (31.2)	
Size 5	68 (21.3)	22 (20.2)	
Size 6	46 (14.4)	15 (13.8)	
Size 7	14 (4.4)	0 (0)	
Size 8	8 (2.5)	2 (1.8)	
Stem fit			0.473**
Distal fit	47 (14.7)	13 (11.9)	
Non-distal fit	273 (85.3)	96 (88.1)	

* Mann-Whitney U test; ** Chi-squared test

*THA* total hip arthroplasty, *BMI* body mass index, *OA* osteoarthritis, *ONFH* osteonecrosis of the femoral head, *PL* postero lateral, *m-WJ* modified Watson-Jones, *DAA* direct anterior approach

### Measurement of femoral size parameters

**Fig. 2:**
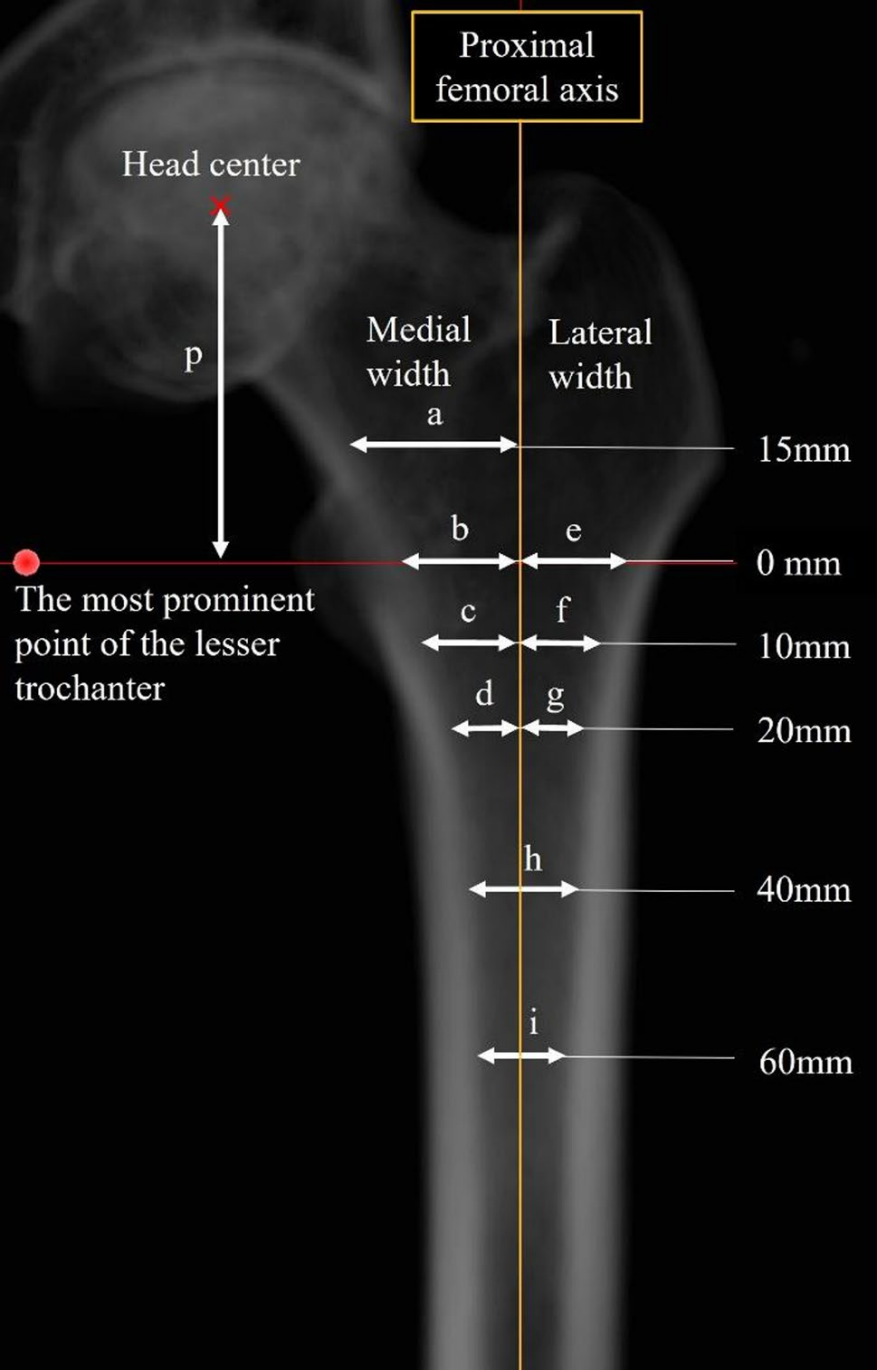
Projection images are created using three-dimensional (3D) computed tomography, and the femoral dimensions are measured at 10 points based on the proximal femoral axis and the most prominent point of the lesser trochanter, which are used as features

For all patients, a femur image projected onto the plane formed by the femoral neck and femoral axes was created using three-dimensional (3D) computed tomography (CT). Ten parameters were measured based on anatomical landmarks, which were used as the feature values for the femoral dimensions (Fig. 2). The measurements included the following:

- The distance from the proximal femoral axis to the medial cortical bone edge at 15 mm proximal to the most prominent point of the lesser trochanter (PLT) was defined as “a.”
- Distances from the proximal femoral axis to the medial cortical bone edge at the PLT, 10 mm distal to the PLT, and 20 mm distal to the PLT were defined as “b,” “c,” and “d,” respectively.
- Distances from the proximal femoral axis to the lateral cortical bone edge at the same locations as mentioned above were defined as “e,” “f,” and “g.”
- The medullary cavity diameters at 40 and 60 mm distal to the PLT were defined as “h” and “i,” respectively.
- Finally, the height of the femoral head center relative to the PLT was defined as “p.”
- Cases were excluded when accurate identification of the femoral head center was not feasible. Specifically, cases in which the ratio of the short axis to the long axis of the projected femoral head was less than two-thirds were excluded.

### Training dataset and feature engineering

The training dataset included femoral size parameters as features, with stem size and compatibility information obtained from simulations as the target variables for supervised learning. Simulations were performed using the 3D templating software ZedHip version 18.0.0 (Lexi Co., Tokyo, Japan). The stem anteversion angle was aligned to match the femoral anteversion angle of the native hip [7], and the center of the femoral head was replicated to closely approximate the native center.

Furthermore, the largest stem size that fits within the proximal femur flare, maintaining a 3° alignment in flexion-extension and varus-valgus directions, was selected. After stem placement, contact was defined as regions with a density of ≥ 543 Hounsfield units [8]. According to Gruen’s classification [9], a fit confined to zones 3, 4, and 5 was classified as a distal fit and deemed a poor fit, whereas a fit in other regions, representing proximal fit, was classified as a good fit.

The two datasets were defined for model development as follows:

1. Baseline dataset: This dataset included the original femoral size parameters as features without any additional variables.
2. Enhanced dataset: This dataset was created by incorporating feature-engineered variables into the baseline dataset. These new variables were derived from combinations of the femoral size parameters, such as ratios that capture structural variations.

**Fig. 3:**
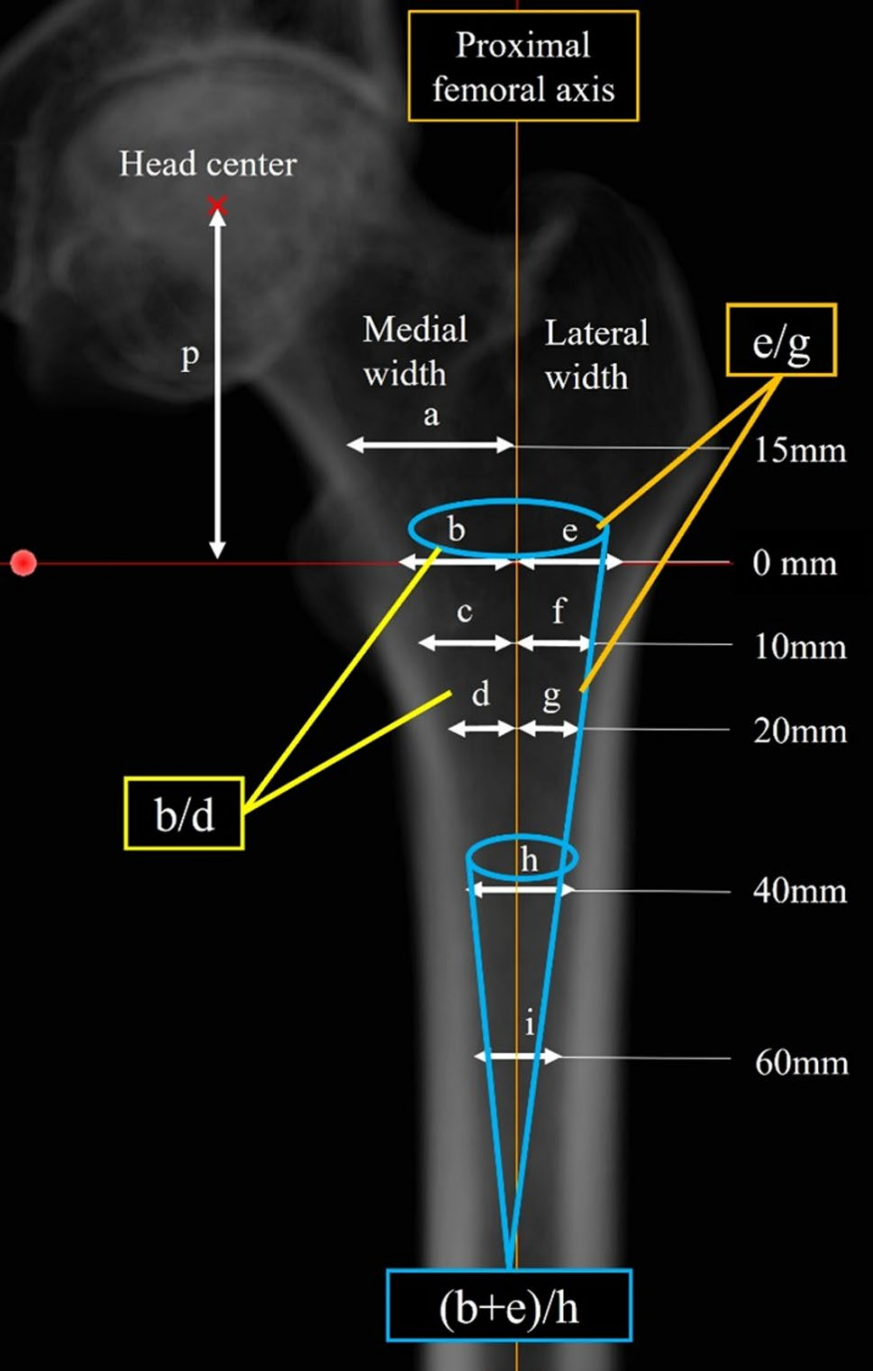
Feature-engineered variables added to the baseline dataset, including ratios that capture structural variations, such as the proximal-to-distal medullary cavity width ratio, to enhance predictions of stem size and compatibility

Feature engineering focused on creating ratios to capture structural variations along the femoral length. These included the proximal-to-distal medullary cavity width ratio (Fig. 3), which provided insights into femoral geometry and aimed to enhance the model’s ability to predict stem size and compatibility. Since the compatibility of taper wedge stems is influenced by the fixation of the proximal femoral flare, specifically the medial “b/d” and lateral “e/g,” as well as the medullary cavity occupancy ratio “(b + e)/h,” these features were added to the enhanced dataset. Features used for synthesis were removed to avoid redundancy. This approach aimed to improve the predictive power by providing a more detailed representation of the femoral structural characteristics.

### Model training

The working environment was set up using Python (version 3.10.12) packages in Google Colaboratory. Preliminary accuracy evaluations were performed using PyCaret (version 3.3.2) to identify the most suitable algorithm for the dataset. The evaluated algorithms included Logistic Regression, K-Nearest Neighbors, Support Vector Machine, Decision Tree, Random Forest (RF), LightGBM, and XGBoost. These models were created using the original dataset derived from the training dataset.

The size prediction task was formulated as a seven-class classification problem, where the goal was to predict the appropriate stem size (from sizes 2 to 8) based on femoral size parameters. In contrast, the compatibility prediction task was defined as a binary classification problem, aiming to determine whether the stem exhibited a distal or proximal fit.

Accuracy was assessed based on exact matches between predicted and actual sizes for size prediction and based on the proportion of correct classifications (distal or proximal fit) for compatibility prediction. Since RF demonstrated the highest accuracy in both tasks, RF was selected for model development (Table 2). Models were implemented using the Scikit-learn library (version 1.2.2).

**Table 2:** Comparison of model accuracies* using PyCaret based on the training dataset

Model	Accuracy	F1 score	Precision	Recall
Size prediction**				
1. Random Forest	0.57	0.55	0.60	0.57
2. Extreme Gradient Boosting	0.56	0.55	0.58	0.56
3. Light Gradient Boosting Machine	0.55	0.54	0.57	0.55
4. K Nearest Neighbors	0.55	0.54	0.57	0.55
5. Logistic Regression	0.53	0.51	0.54	0.53
6. Decision Tree	0.52	0.52	0.55	0.52
7. Support Vector Machine	0.39	0.37	0.39	0.39
Fit prediction				
1. Random Forest	0.80	0.76	0.76	0.80
2. Extreme Gradient Boosting	0.79	0.76	0.75	0.79
3. Light Gradient Boosting Machine	0.79	0.76	0.76	0.79
4. Logistic Regression	0.76	0.72	0.69	0.76
5. K Nearest Neighbors	0.75	0.72	0.72	0.75
6. Decision Tree	0.72	0.72	0.73	0.72
7. Support Vector Machine	0.72	0.68	0.67	0.72

Ranked in order of highest accuracy

*Obtained using the original dataset during the validation phase, **Correct if margin of error is ± 0

Two models were developed for size and compatibility predictions. For each model type, two versions were created using the datasets defined in Sect.  2.4 as follows: (1) the Baseline model, trained with the baseline dataset containing the original femoral size parameters alone, and (2) the Enhanced model, trained with the enhanced dataset, which included both the original parameters and feature-engineered variables.

Data preparation included standardization and the application of the synthetic minority oversampling technique [10] to address class imbalance. The standardization coefficients calculated from the training data were applied to the test data. Specifically, the baseline and enhanced datasets were each split into an 80% training set and a 20% validation set for model training and accuracy validation.

### Test dataset and model accuracy evaluation

The test dataset comprised patients different from those included in the training datasets (baseline and enhanced datasets). Additionally, the ground truth was defined as the stem size used during surgery and the fit evaluated from the postoperative anteroposterior hip radiograph.

Both the Baseline and Enhanced models were applied to the test dataset, and their accuracies were validated. The accuracy of the size prediction model was assessed with a tolerance of approximately ± 1 size difference between the predicted and actual stem sizes. Exact-match accuracy was used during model development for strict comparison across algorithms, whereas a ± 1 size tolerance was adopted in the test evaluation to better reflect clinical practice, where preoperative planning is typically used to estimate a range of appropriate implant sizes. For the compatibility prediction model, exact matches between the predicted and actual fit classifications (distal or proximal fit) were used to evaluate accuracy.

The evaluation metrics included accuracy, F1 score, precision, and recall. Feature importance analysis was used to assess the contribution of each feature to the model’s predictions. All statistical analyses were performed using IBM SPSS Statistics for Windows, version 25 (IBM Corp., Armonk, NY, USA), with statistical significance set at *p* < 0.05.

## Results

**Fig. 4:**
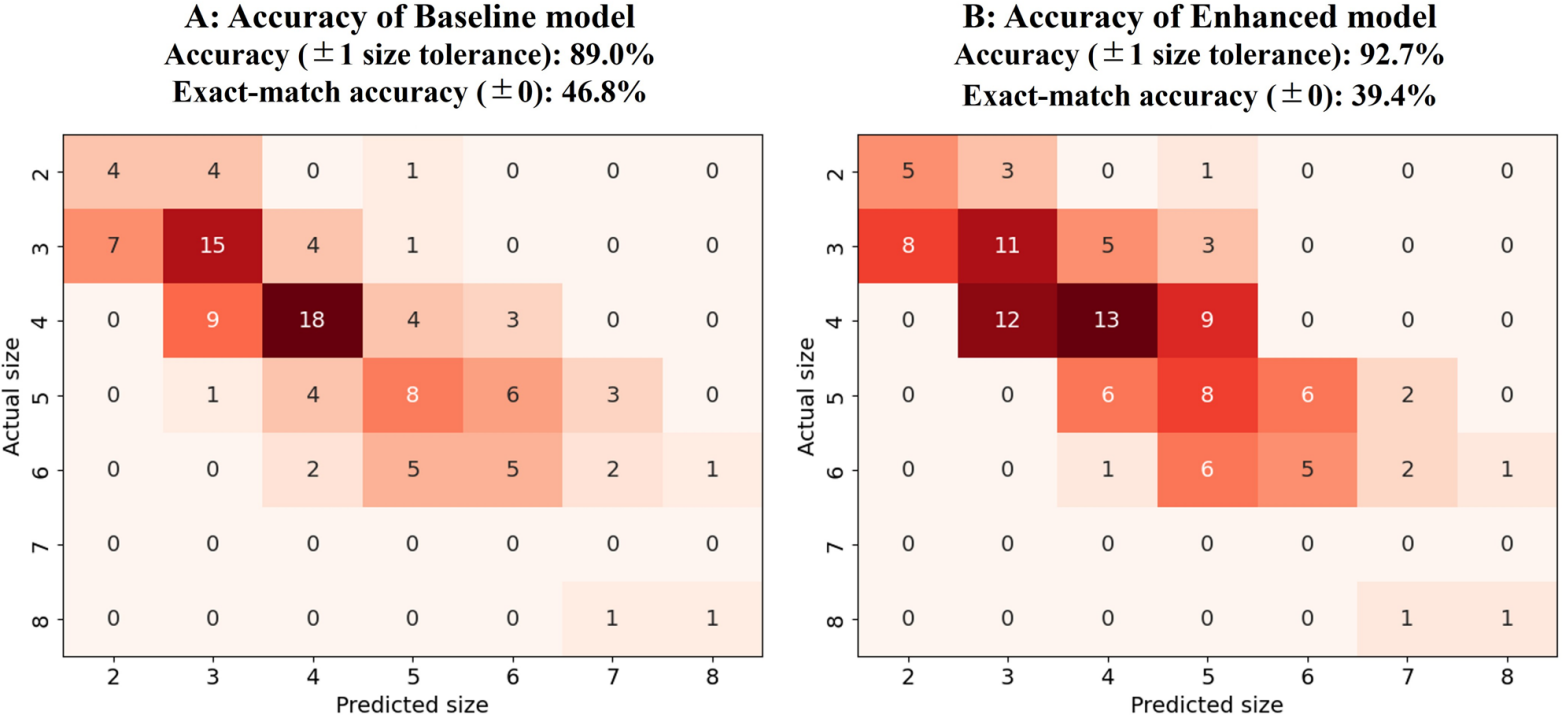
Accuracy and confusion matrix for size prediction in each model. The size prediction accuracy of the Baseline model **(A)** was compared with that of the Enhanced model **(B)**. Specifically, the inclusion of feature-engineered variables in the dataset resulted in a 3.7% improvement in accuracy

The size prediction accuracy was 89.0% (F1 score, 0.755; precision, 0.753; and recall, 0.769) and 92.7% (F1 score, 0.781; precision, 0.782; and recall, 0.793) for the Baseline and Enhanced models, respectively. Specifically, the Enhanced model demonstrated fewer two-size errors than the Baseline model, resulting in higher overall accuracy (Fig. 4). The exact-match accuracy (± 0), representing a stricter evaluation criterion, was 46.8% (F1 score, 0.393; precision, 0.400; and recall, 0.389) and 39.4% (F1 score, 0.360; precision, 0.368; and recall, 0.363) for the Baseline and Enhanced models, respectively.

**Fig. 5:**
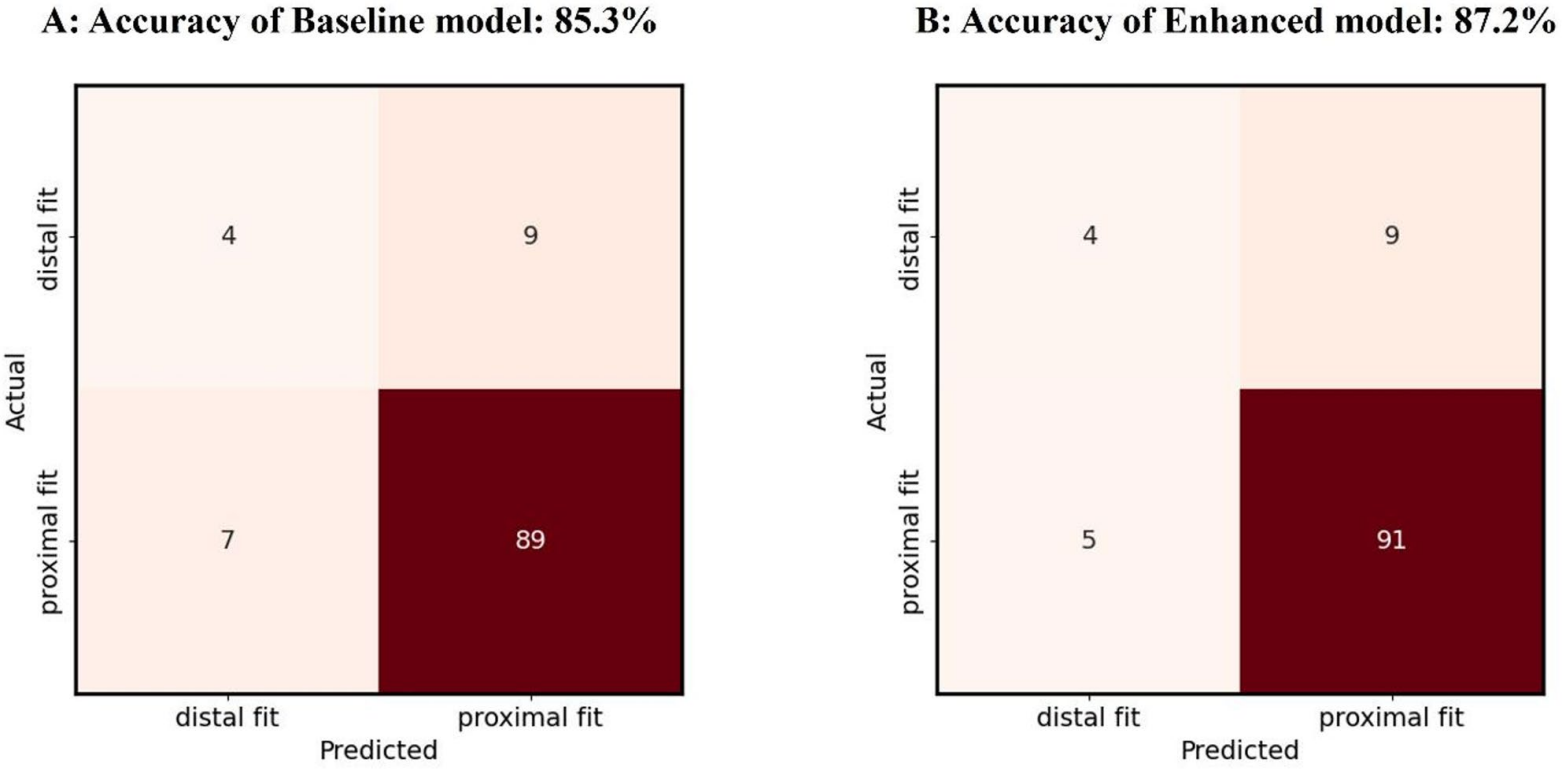
Accuracy and confusion matrix for fit prediction in each model. The fit prediction accuracy of the Baseline model **(A)** was compared with that of the Enhanced model **(B)**. The inclusion of feature-engineered variables in the dataset resulted in a 1.9% improvement in accuracy

The compatibility prediction accuracy was 85.3% (F1 score: 0.918, precision: 0.908, and recall: 0.927) and 87.2% (F1 score: 0.929, precision: 0.910, and recall: 0.948) for the Baseline and Enhanced models, respectively (Fig. 5).

**Fig. 6:**
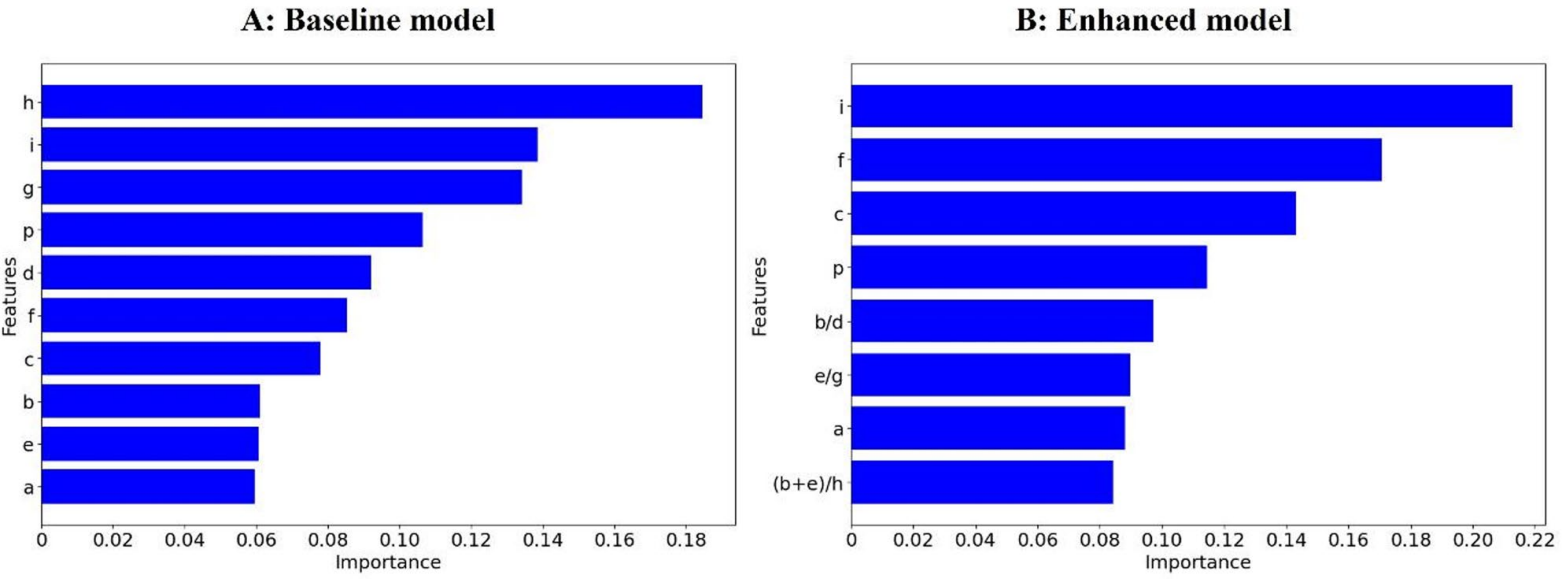
Feature importance for the size prediction model, highlighting the critical role of distal femoral dimensions. The Baseline model identified the distal medullary cavity diameter (h) as the most influential feature **(A)**, while the Enhanced model replaced it with the distal medullary cavity diameter (i) as the most influential feature. However, the distal medullary cavity diameter remained the most critical parameter overall **(B)**

Feature importance analysis provided insights into the factors influencing the predictions for the size and compatibility models. For the size prediction model (Fig. 6), the Baseline model identified the distal medullary cavity diameter (h) as the most influential feature. In the Enhanced model, while the distal medullary cavity diameter (i) replaced h as the top contributor, the distal medullary cavity diameter remained the most critical parameter overall. Additionally, engineered variables such as b/d and e/g emerged among the top-ranked features, demonstrating the added values of incorporating composite metrics that capture the relationship between proximal and distal femoral dimensions.

**Fig. 7:**
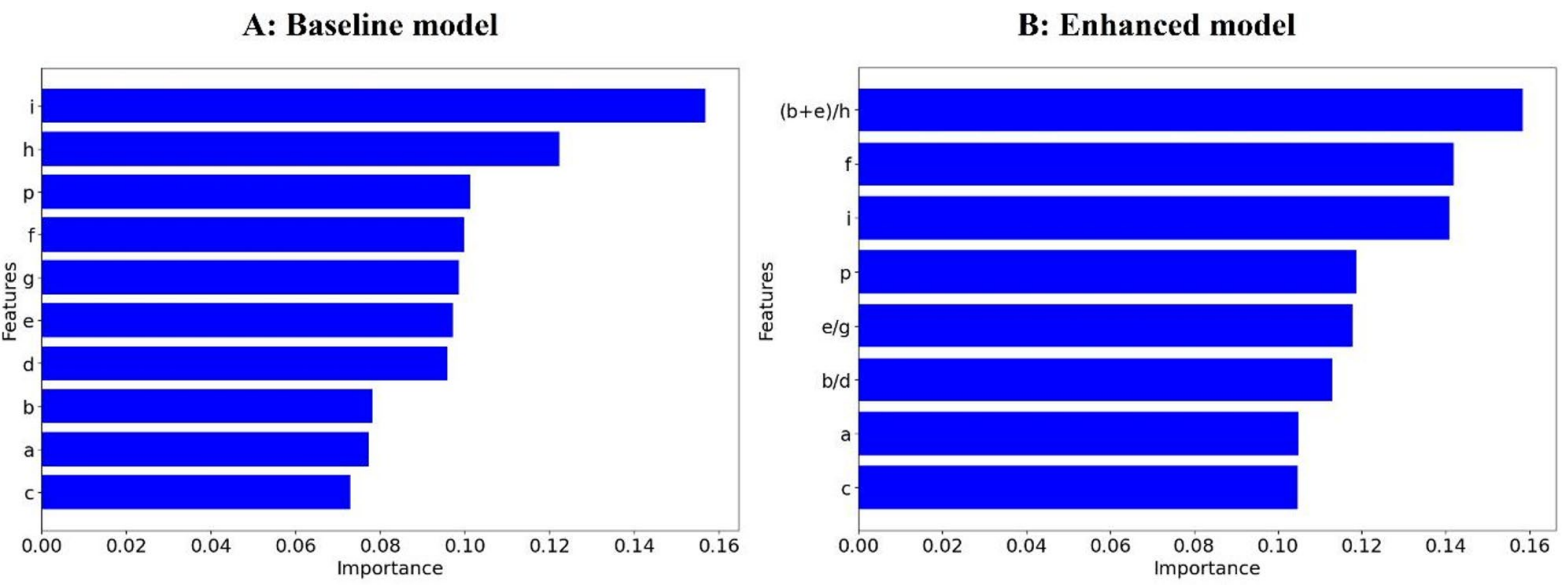
Feature importance for the compatibility prediction model, emphasizing distal femoral dimensions (i and h) in the Baseline model **(A)**. The Enhanced model identified (b + e)/h as the most influential feature, along with e/g and b/d, all of which capture proximal-distal femoral relationships **(B)**

For the compatibility prediction model (Fig. 7), the Baseline model relied heavily on the distal medullary cavity diameters (i and h) but also distributed importance across various femoral size parameters. However, the Enhanced model showed a notable shift, with engineered ratios such as (b + e)/h emerging as the most influential feature.

## Discussion

In this study, machine learning models were developed to predict stem size and compatibility based on the femoral dimensions derived from simulation results used as training data. The Enhanced model achieved a stem size and compatibility prediction accuracy of 92.7% and 87.2%, respectively. For size prediction, the distal medullary cavity diameter was the most critical feature, whereas the importance was distributed across features for compatibility prediction. The Enhanced model, which incorporated feature-engineered variables, demonstrated a 3.7% improvement in size prediction accuracy compared to the Baseline model. These findings have important clinical implications. The proposed models may support surgeons in preoperative planning by providing objective estimations of stem size and compatibility based on femoral geometry. This approach may help standardize decision-making, reduce inter-surgeon variability, and assist less experienced surgeons or institutions without advanced planning tools.

The size prediction accuracy of the models was comparable to that of 3D planning methods based on the features extracted from two-dimensional (2D) projection images. Previous reports on preoperative stem size prediction have shown accuracies ranging from 61% to 83% and 84% to 98% for 2D and 3D planning, respectively; the accuracies of our models were comparable to those of 3D planning (Table 3) [11–15]. This high accuracy of our models can be attributed to the use of 3D simulation results as training data, which provided a detailed representation of femoral geometry. Although the differences in accuracy among the evaluated models were small, Random Forest was selected considering its robustness to overfitting, stable performance, and interpretability through feature importance, as well as its suitability for clinical implementation with relatively low computational requirements.

**Table 3:** Comparison of stem size prediction accuracy between 2D and 3D templating methods

Study	Hips	Templating methods	Exact (± 0)	± 1 size
Brenneis et al. [11]	23	2D: digital templating	35.7%	60.7%
	28	3D: EOS based software	34.8%	91.3%
Mainard et al. [12]	31	2D: acetate templating	32%	68%
	31	3D: EOS based software	34%	84%
Schiffner et al. [13]	116	2D: digital templating	45.7%	83.6%
	116	3D: CT based software	58.6%	94%
Huo et al. [14]	59	2D: digital templating	49.2%	74.6%
	59	3D: CT based software	76.3%	93.2%
Viceconti et al. [15]	29	2D: acetate templating	34%	83%
	29	3D: CT based software	52%	86%
Present study				
Baseline model	109	Random Forest model	46.8%	89%
Enhanced model	109	Random Forest model	39.4%	92.7%

The clinical interpretation of the ± 1 size tolerance used in this study warrants further discussion. In clinical practice, preoperative templating is primarily used to estimate an appropriate range of implant sizes, and discrepancies of approximately ± 1 size between the planned and implanted stem are not uncommon. The final implant size is routinely confirmed intraoperatively through trial and direct assessment. From a clinical perspective, under-sizing may result in insufficient fixation, whereas oversizing may increase the risk of intraoperative fracture or excessive cortical stress. However, these risks are generally mitigated in routine surgical workflows through intraoperative evaluation and adjustment. Therefore, the proposed model should be interpreted as a decision-support tool that assists in narrowing the range of candidate stem sizes, rather than as a system providing definitive recommendations. This approach may contribute to improving the efficiency and consistency of preoperative planning while preserving the role of surgical judgment in final implant selection.

Our study demonstrated that creating a highly accurate prediction model for the target stem is possible using simulation data as training data, relying solely on hip CT data. The accuracy of our model was within the range of values reported in previous studies using deep learning-based preoperative planning systems with image recognition, which reported an accuracy of 95.5% within ± 1 size tolerance [16]. Furthermore, it can predict compatibility, which is as important as size in determining stem suitability. Deep learning approaches, while adept at capturing complex relationships, typically require substantially larger datasets and are prone to overfitting when training data are limited [6]. In contrast, RF classification models process numerical data [17, 18] and perform well with smaller datasets. Therefore, this study streamlined the model creation process by transforming femoral dimensions from images into numerical features, enabling the development of RF models with robust generalization capabilities.

Feature importance analysis revealed that the distal medullary cavity diameter was crucial for size prediction, whereas compatibility prediction depended on a broader range of femoral dimensions. This tendency was similar to surgeons’ decision-making processes during preoperative planning, where stem size is selected based on medullary cavity occupancy, and the fit is assessed by examining contact points between the stem and bone. The ratios between the proximal and distal medullary cavity widths, as well as the compatibility with stem morphology, are also critical factors. By introducing these ratios, the Enhanced model effectively captured relative relationships between different regions of the femur, providing a more comprehensive representation of the geometry. This enabled the model to detect subtle differences in structural characteristics, thereby improving the accuracy of both size and compatibility predictions. The inclusion of feature-engineered variables, such as these ratios, in the Enhanced model likely contributed to its improved accuracy by better capturing complex geometric relationships. Another possible interpretation for this improvement is that feature engineering facilitates the creation of boundaries in multidimensional parameter space, which effectively separate data points distributed within the space. Consequently, these boundaries enhance model training efficiency by making the data more separable [19].

Integrating the prediction models developed in this study with automated feature extraction from images could enable the automation of preoperative planning. Although some studies have reported on the extraction of bone dimensions using deep learning-based models [20], further improvement in accuracy is needed. We are currently exploring edge detection methods, which may provide the basis for developing fully automated preoperative planning systems in the future.

This study has some limitations. First, selection bias may have been introduced in this study. Cases with severe femoral head deformation were excluded because accurate identification of the femoral head center was not feasible, which may limit applicability to clinically challenging cases where preoperative planning is particularly important. In addition, the study population consisted of Asians, which may restrict the generalizability of the findings to other populations. Furthermore, selection bias in femoral morphology may exist between cases using the Accolade II stem and those using other stem types, as the former were reserved for external validation and excluded from the training dataset. Addressing these issues will require further studies incorporating more diverse patient populations and implant types. Second, stem compatibility was defined using a binary classification based on fixation pattern relative to the porous-coated region of the Accolade II stem. Although this approach simplifies the assessment of exact stem–bone fit, it may not fully capture the complexity of implant–bone interactions, despite reflecting a clinically relevant distinction between proximal and distal fixation. Third, there may be inconsistency between the simulation-based training labels and the radiographic test labels. As the training data were derived from CT-based simulations and the test data from radiographic evaluation, discrepancies may occur depending on imaging conditions. However, because compatibility was defined as a binary classification (distal vs. non-distal fixation), the impact of this difference is considered to be limited, although it remains a potential source of bias. Fourth, different stem types were used in the training and test datasets, reflecting variations in implant selection over time and among surgeons. Although this variation was primarily due to surgeon preference, it may affect the generalizability of the model. Therefore, the present model should be interpreted as being validated for the Accolade II stem, and further studies are needed to extend its applicability to other implant systems. Fifth, the training data was based on simulation results obtained by a single examiner. Integrating the results from multiple examiners could potentially improve the accuracy; however, its impact is expected to be minimal since the simulation protocol was thoroughly reviewed. Finally, other potentially important features that were not used in this study may exist. Although the model demonstrated good performance, further refinement of the feature set could enhance predictive performance. Feature engineering contributed to improving the model’s accuracy in this study and is likely to play a pivotal role in achieving even higher performance in future models.

## Conclusion

The models developed in this study may support preoperative planning for total hip arthroplasty using the Accolade II stem by enabling objective estimation of stem size and compatibility. While further validation is required, this approach may contribute to improving the consistency and efficiency of stem selection. However, this evaluation was performed in patients undergoing total hip arthroplasty using the Accolade II stem, and the applicability of the model to other implant systems remains to be established.

## Data Availability

The data utilized in this study is securely stored at the Department of Orthopaedic Surgery, Yamaguchi University Graduate School of Medicine. Datasets created and/or analyzed during the study can be provided upon reasonable request to the corresponding author. For further inquiries, please feel free to contact the corresponding author.

## Abbreviations

RF: Random Forest
THA: total hip arthroplasty
3D: three-dimensional
PLT: point of the lesser trochanter
2D: two dimensional
CT: computed tomography
